# Is the temporomandibular joint affected by rheumatoid arthritis? A comparative investigation with knee arthritis in an experimental rat model

**DOI:** 10.1111/joa.70080

**Published:** 2025-12-07

**Authors:** Ana Carolina de Figueiredo Costa, Luane Macedo de Sousa, Delane Viana Gondim

**Affiliations:** ^1^ Postgraduate Program in Dentistry, Faculty of Pharmacy, Dentistry and Nursing Federal University of Ceará Fortaleza CE Brazil; ^2^ Postgraduate Program in Morphofunctional Sciences, Faculty of Medicine Federal University of Ceará Fortaleza CE Brazil

**Keywords:** articular damage, comparative anatomy, knee, pain, rheumatoid arthritis, temporomandibular joint

## Abstract

Rheumatoid arthritis (RA) is an autoimmune disease that primarily affects hyaline cartilage, except in the case of the temporomandibular joint (TMJ), which is covered by fibrocartilage. This study compared the progression of RA in these two types of cartilage by examining the TMJ and knee joints of rats in both the acute and chronic phases of the disease. Forty‐eight male Wistar rats were divided into groups: Control (animals without RA in the TMJ or knee), RA‐24h, and RA‐7d (animals with RA in the TMJ or knee, euthanized 24 h or 7 days after the last intra‐articular injection, respectively). The rats were sensitized with subcutaneous injections containing complete/incomplete Freund's adjuvant and methylated bovine serum albumin (mBSA), followed by three intra‐articular mBSA injections (one per week) into either the TMJ or knee. Euthanasia was performed 24 h (acute phase) or 7 days (chronic phase) after the third injection. The following parameters were assessed: nociceptive thresholds, cellular influx in synovial fluid, histopathology, immunohistochemistry for metalloproteinase‐9 (MMP‐9), and birefringence of collagen fibers in articular cartilage. A significant reduction in the nociceptive threshold was observed in arthritic animals in both joints compared to the control groups. Additionally, a significant increase in cellular influx in the synovial membrane was noted in both joints after the third mBSA injection, as well as in the knee after 7 days. Histopathological analysis revealed reduced metachromasia, increased MMP‐9 immunoexpression, and higher levels of type III collagen in the articular cartilage compared to the respective controls (*p* < 0.05). The nociceptive response was similar in both joints during the acute and chronic phases of RA. However, evidence of articular repair was observed in the TMJ, accompanied by a sustained reduction in the nociceptive threshold, suggesting central sensitization without ongoing peripheral damage.

## Graphical abstract

Animals were sensitized with mBSA emulsified in CFA/IFA and subsequently received three intra‐articular injections of mBSA into either the TMJ or the knee (once per week). Mechanical hyperalgesia was assessed using electronic von Frey testing after arthritis establishment at 24 h or 7 days following the third intra‐articular injection. Nociceptive thresholds decreased in both joints at the evaluated time points. Synovial fluid and articular tissue analyses revealed joint‐specific inflammatory patterns: knee arthritis showed a sustained increase in all inflammatory parameters, accompanied by persistent joint damage at both time points. In contrast, TMJ inflammation displayed a distinct temporal profile. The acute phase (24 h) was characterized by an increased cellular influx in the synovial membrane, elevated inflammatory parameters, enhanced type III collagen, and joint damage. By 7 days (chronic phase), there was a reduction in inflammatory parameters and signs of joint repair; however, nociceptive thresholds remained low, suggesting the development of central sensitization.

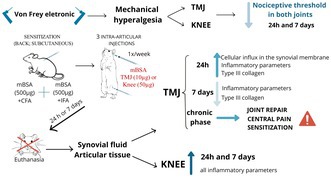

## 1 INTRODUCTION

Rheumatoid arthritis (RA) is one of the most common systemic autoimmune diseases, with a worldwide prevalence of 0.5% to 1% (Almutairi et al., Almutairi et al., 2021; Smolen et al., 2010). It is a chronic and progressive condition that can affect synovial joints as well as extra‐articular organs, including the heart, kidneys, lungs, digestive system, eyes, skin, and nervous system (Conforti et al., 2021; Radu & Bungau, 2021).

The progression of RA is characterized by fluctuations between periods of disease exacerbation and remission (Chaurasia et al., 2020). In the absence of early intervention, symptoms gradually worsen, leading to irreversible joint damage and significantly impairing the physical and emotional well‐being of affected individuals (Fraenkel et al., 2021; Radu & Bungau, 2021). Additionally, complications and comorbidities associated with RA can reduce life expectancy (Lassere et al., 2013).

In the pathogenesis of RA, autoantibodies and immune cells infiltrate and accumulate within the synovial cavity, causing arthralgia, proliferation of synoviocytes and fibroblasts, formation of pannus, activation of matrix metalloproteinases (MMPs) that degrade the collagen matrix, destruction of cartilage and underlying bone, and, ultimately, joint deformity (Kurowska et al., 2017). Initially, the small joints of the hands and feet are typically affected bilaterally (Scherer et al., 2020).

The temporomandibular joint (TMJ) is a synovial joint, but it differs morphologically and physiologically from other joints (Lemos et al., 2018; Vos et al., 2014). TMJ cartilage consists of four layers: the articular zone, the zone of undifferentiated cells, the flat zone, and the hypertrophic zone. It is primarily composed of fibrocartilage, with a predominance of type I collagen fibers, and has a greater capacity for adaptation and repair due to its unique anatomical and histological structure (Chandrasekaran et al., 2021; Fazaeli et al., 2016). In contrast, other synovial joints, such as the knee, are lined with hyaline cartilage, composed of two layers, a predominance of type II collagen fibers, and are more susceptible to damage (Gutman et al., 2018). Moreover, the functional mechanics of these two joints differ. The knee joint operates along a single axis, defined by the fixation of tendons, muscles, and structural components (Kubiak & Ditzel, 2016). In contrast, the TMJ is a bicondylar joint, which facilitates both hinge and sliding movements. This is made possible by the interaction between the disc, the mandibular fossa of the temporal bone, and the surrounding connective tissue, allowing movement along a single axial plane (Nazet et al., 2022).

Given these differences, the development of experimental models of joint inflammation is crucial for understanding the molecular mechanisms and unique responses of each joint under pathological conditions. Therefore, the aim of this study was to conduct a comparative analysis of an experimental model of arthritis in the TMJ and knee of rats during both the acute and chronic phases of the disease.

## 2 MATERIALS AND METHODS

### 2.1 Animals

The experimental protocols were approved by the Animal Use Ethics Committee (CEUA) of the Federal University of Ceará (number 4747280219) and followed the recommendations of the National Research Council and the National Institute of Health. Additionally, the guidelines established by the ARRIVE statement were followed.

Forty‐eight male Wistar rats (7–8 weeks old; 180–220 g; three animals per cage) were provided by the Central Animal Facility of the Federal University of Ceará and maintained in polypropylene cages, controlled temperature (25 ± 2°C), light/dark cycle of 12 h/12 h, relative humidity of 60% and free access to water and food.

The animals were randomly divided into the following experimental groups (*n* = 6), using a computer‐based random order generator: Control (animals received sterile 0.9% saline solution in the TMJ (TMJ/C) or knee (K/C) and were euthanized 24 h or 7 days after the 3rd intra‐articular injection); RA‐24h (animals received mBSA injections in the TMJ (TMJ/RA‐24h) or knee (K/RA‐24h) and were euthanized 24 hours after the third intra‐articular injection); RA‐7d (animals received mBSA injections in the TMJ (TMJ/RA‐7d) or knee (K/RA‐7d) and were euthanized 7 days after the third intra‐articular injection).

The sample size was determined based on the study by de Sousa et al. (2019) with the objective of identifying as significant a difference of 20% between the groups, statistical power of 80% and standard deviation set at 15% within a 95% confidence interval. The percentage of animals with arthritis induced by mBSA is 80%. As a result, six animals were included in each experimental group.

### 2.2 RA induction

The animals were initially sensitized with a subcutaneous injection on the back containing 500 μg of methylated bovine serum albumin (mBSA; Sigma‐Aldrich, Missouri, USA) diluted in an emulsion of 100 μL phosphate‐buffered saline (PBS) and 100 μL Complete Freund's Adjuvant (CFA; Sigma‐Aldrich, Missouri, USA). Subcutaneous booster injections, containing 500 μg of mBSA, 100 μL PBS, and 100 μL Incomplete Freund's Adjuvant (IFA; Sigma‐Aldrich, Missouri, USA), were administered 7 and 14 days later at different locations on the animals' backs (Quinteiro et al., 2014).

After inducing intraperitoneal anesthesia with 2% ketamine (80 mg/kg) and 10% xylazine (10 mg/kg), intra‐articular injections were administered to the rats' left TMJ or knee (10 or 50 μg of mBSA dissolved in 10 or 50 μL PBS, respectively) 21 days after the beginning of the experimental protocol. Intra‐articular booster injections of mBSA were then administered on days 28 and 35 (de Sousa et al., 2019, adapter from Chandrupatla et al.). Control groups received intra‐articular injections of saline solution into the TMJ or knee on days 21, 28, and 35. The animals were euthanized 24 h or 7 days after the third intra‐articular booster injection using an overdose of 10% ketamine and 2% xylazine, on days 36 and 42, respectively (Supplementary Material S1).

### 2.3 Assessment of mechanical hyperalgesia in the TMJ and knee

Mechanical hyperalgesia was assessed using the electronic von Frey test. The animals were conditioned to the test for 5 days under controlled temperature and reduced lighting conditions. For the test, the animals were individually placed in polysulfone cages for 20 min and then subjected to the application of a gradual, perpendicular force to the region of the left TMJ or left knee until a reflex response was observed (head or paw withdrawal). This application was repeated three times, and the force intensity (in g) at which the response occurred was recorded. The recorded values were then averaged (de Sousa et al., 2019; de Sousa et al., 2022; Gusmão et al., 2021). The tests were performed by a calibrated evaluator who was blinded to the group assignments on days 20, 21, 24, 27, 28, 31, 34, 35, 36, and 42. On the days when intra‐articular injections were performed, mechanical hyperalgesia was assessed 6 h after the administration of mBSA into the joint of interest.

### 2.4 Assessment of cellular influx in synovial fluid of the TMJ or knee

After euthanasia, the skin and muscles around the TMJ or knee were dissected. A 30G needle was inserted through the synovial membrane, and the synovial cavity was washed by injecting and immediately aspirating 50 μL of PBS supplemented with 10 mM ethylenediaminetetraacetic acid (EDTA). This washing procedure was repeated, and synovial fluid was collected. Aliquots of 20 μL of synovial fluid were diluted in 380 μL of Türk's solution. A calibrated examiner determined the total leukocyte count using a Neubauer chamber hemocytometer (BRAND®, Sigma‐Aldrich, Missouri, USA) and an optical microscope (Leica DM 2000, Wetzlar, Germany). The results were expressed as the number of cells in the synovial fluid (×10^6^) (Camargo et al., 2021).

### 2.5 Histopathological evaluation of the TMJ and knee

The left TMJ or knee joints were dissected, fixed in 10% buffered formalin for 48 h, and demineralized in a 10% EDTA suspension (pH 7.3, NaOH, PA) for four consecutive weeks. The samples were embedded in paraffin blocks, cut into 5 μm sections, and mounted on glass slides. Hematoxylin–eosin and 0.4% toluidine blue stains were used for the descriptive histopathological evaluation of the synovial membrane (SM) and articular cartilage (AC). The sections were dehydrated using increasing concentrations of ethanol and xylene, mounted on glass slides, and examined under an optical microscope (Leica DM 2000, Wetzlar, Germany). An experienced pathologist, blinded to the experimental groups, analyzed the samples (de Sousa et al., 2023; Zhang et al., 2018).

For the semiquantitative analysis, an adapted version of a previously described scoring method was used (Tak et al., 1997). The score was based on the inflammatory infiltrate in the SM (0 = normal; 1 = mild inflammatory infiltrate; 2 = moderate inflammatory infiltrate; and 3 = intense inflammatory infiltrate). The destruction of the AC and subchondral bone was assessed on a scale of 0–3 (0 = normal; 1 = mild cartilage destruction; 2 = evidence of cartilage and bone destruction; and 3 = severe cartilage and bone destruction). Pannus formation was scored as 0 (absence of pannus) or 1 (presence of pannus).

### 2.6 Analysis of collagen birefringence in the articular cartilage of the TMJ and knee

The TMJ or knee samples were incubated in 0.1% picrosirius red (ScyTek®) for 30 min, washed in 5% hydrochloric acid, stained with Harris hematoxylin, and mounted with Enhtellan®. The slides were analyzed using a polarized light microscope (Leica DM 2000, Wetzlar, Germany), where type I collagen appeared red‐orange and type III collagen appeared greenish‐white.

To quantify collagen types I and III, five fields at 400× magnification were photographed using a camera attached to the polarized light microscope. Collagen birefringence was then evaluated by analyzing the photomicrographs with ImageJ software.

### 2.7 MMP‐9 immunoexpression in the articular cartilage of the TMJ or knee

The immunohistochemical staining for matrix metalloproteinase‐9 (MMP‐9) in the TMJ and knee was performed using the streptavidin‐biotin‐peroxidase method. Samples were cut into 4 μm sections, mounted on poly‐L‐lysine‐coated slides, deparaffinized, rehydrated, and subjected to antigen retrieval with citrate buffer (DAKO, pH 6.0) at 85°C for 30 min using PT Link (DAKO; Santa Clara, CA, United States). Endogenous peroxidase activity was blocked by incubating the sections with 3% (v/v) hydrogen peroxide for 20 min at room temperature, followed by washing in PBS.

Next, the sections were incubated with the primary antibody against MMP‐9 (Santa Cruz, H‐129, 1:200) diluted in antibody diluent solution (DAKO) for 1 h at room temperature. After washing with buffer, the sections were incubated with HRP‐conjugated polymer (DAKO; Santa Clara, CA, United States) for 30 min. The slides were then washed, stained with the chromogen 3,3'‐diaminobenzidine (DAB), counterstained with Mayer's hematoxylin, dehydrated in a graded ethanol series, cleared with xylene, and coverslipped. Negative controls were processed in parallel using the same protocol, except the primary antibody was replaced with antibody diluent.

Images were captured using an optical microscope equipped with a camera and acquisition system (Leica DM 2000, Wetzlar, Germany). Five fields per slide (400× magnification) were selected from areas with the highest staining intensity (“hot spots”) to count chondrocytes exhibiting cytoplasmic and/or nuclear positivity (brown staining) for MMP‐9. The average count of these fields was then calculated (de Sousa et al., 2023).

### 2.8 Statistical analysis

The normality of the data distribution was assessed using the Shapiro–Wilk test. Parametric data were analyzed using one‐way ANOVA followed by Tukey's post hoc test, or two‐way mixed repeated‐measures ANOVA followed by Tukey's post hoc test when appropriate. Nonparametric data were analyzed using the Kruskal–Wallis test, and when significant, Dunn's post hoc test was applied for multiple group comparisons. Data are presented as mean ± standard deviation (SD) for parametric variables, and as median (minimum–maximum) for nonparametric variables. Statistical analyses were performed using GraphPad Prism version 7.0 (GraphPad Software, La Jolla, CA, USA). A *p* <0.05 was considered statistically significant.

## 3 RESULTS

### 3.1 mBSA‐induced arthritis in the TMJ or knee increases nociceptive response

**FIGURE 1:**
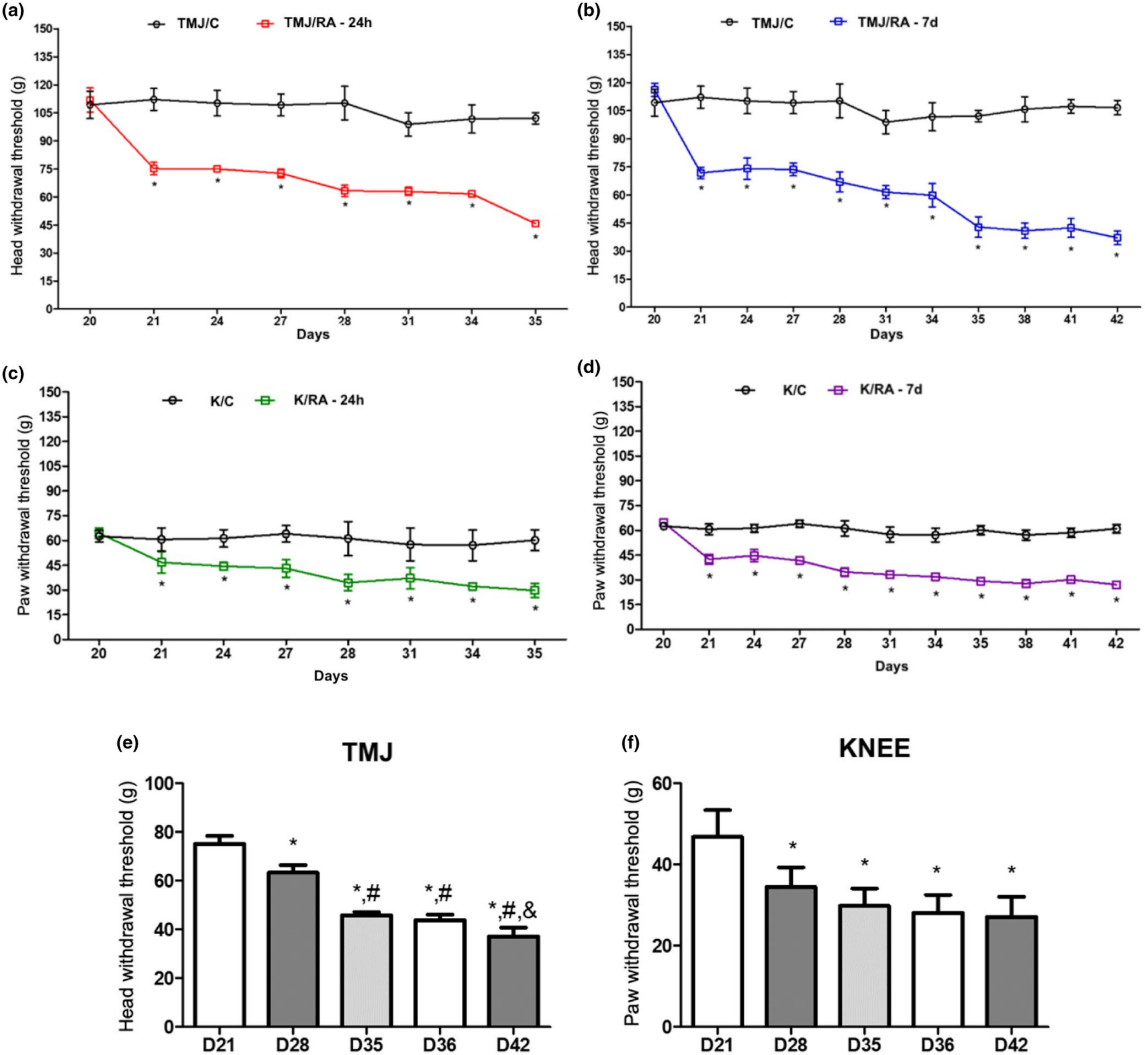
Reduction in nociceptive threshold in rats (*n* = 6/ group) with mBSA‐induced arthritis in the TMJ and knee. Mechanical hyperalgesia was evaluated throughout arthritis development up to 6 h (a and c) and up to 7 days (b and d) after the third intra‐articular injection in the TMJ and knee. Results are presented as mean ± SD (*p < 0.05; two‐way repeated‐measures ANOVA, Tukey). The nociceptive response of arthritic animals was also analyzed in the TMJ (e) and knee (f) up to 42 days after the start of the experimental protocol. Results are presented as mean ± SD (*p < 0.05; one‐way ANOVA, Tukey) *p < 0.05 versus D21; ^#^ *p* < 0.05 versus D28; & versus D35 and D36.

The nociceptive threshold of arthritic animals was significantly reduced in both joints and across different euthanasia time points compared with control groups (*p* < 0.0001) (Figure 1a–d).

In the TMJ, booster injections promoted a sustained reduction in nociceptive threshold over time compared with the corresponding control time points (*p* < 0.0001) and the baseline values of arthritic animals. Mixed ANOVA revealed a significant effect of time (*F*_1,32_ = 900.5; *p* < 0.0001) and a significant group × time interaction (*F*_7,32_ = 25.83; *p* < 0.0001). A significant reduction in nociceptive threshold was detected between D21 and D28 (*p* = 0.013), D35 (*p* < 0.0001), D36 (*p* < 0.0001), and D42 (*p* < 0.0001). Following the second booster injection, a decrease in nociceptive threshold was observed, but no significant differences were found compared with D31 and D34. However, after the third intra‐articular injection, nociceptive responses were significantly higher compared with D28 (*p* < 0.0001) and D42 (*p* = 0.002) (Figure 1e).

For the knee joint, mixed ANOVA showed a significant effect of time (*F*_1,32_ = 143.2; *p* < 0.0001) and a significant group × time interaction (*F*_7,32_ = 4.912; *p* < 0.0001). A significant difference was observed between baseline (D20) and all subsequent time points (*p* < 0.0001) (Figure 1c,d). Nociceptive responses were significantly higher 6 h after the first booster injection (D21) compared to baseline (D20). A significant reduction in nociception was observed following the third booster injection compared to D21. However, no significant difference was detected between the second (D28) and third (D35) boosters, nor between 6 h and 7 days after the third booster injection (Figure 1f). No significant differences were observed over time in the control group.

### 3.2 m‐BSA‐induced arthritis in the TMJ increases cellular influx and inflammatory parameters only in the acute phase

**FIGURE 2:**
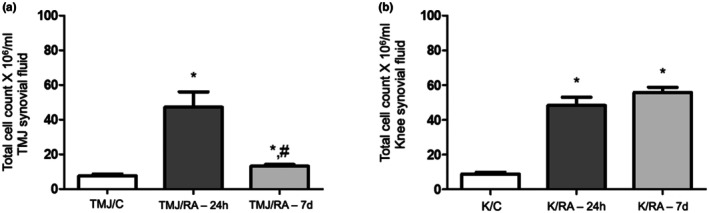
Total cell count in the TMJ (a) and knee joint (b) 24 h or 7 days after the third intra‐articular injection of mBSA (*n* = 6/ group). Results are presented as mean ± SD (One‐way ANOVA, Tukey). *p < 0.05 versus TMJ/C or K/C; #*p* < 0.05 versus TMJ/RA‐24h. TMJ = temporomandibular joint. TMJ/C = Control group; animals that did not receive mBSA in the TMJ. TMJ/RA‐24h = TMJ arthritic animals euthanized 24 h after the third intra‐articular injection of mBSA. TMJ/RA‐7d = TMJ arthritic animals euthanized 7 days after the third intra‐articular injection of mBSA. K/C = Control group; animals that did not receive mBSA in the knee joint. K/RA‐24h = Knee arthritic animals euthanized 24 h after the third intra‐articular injection of mBSA. K/RA‐7d = Knee arthritic animals euthanized 7 days after the third intra‐articular injection of mBSA.

Cellular influx into the synovial fluid increased significantly in the TMJ/RA‐24h and K/RA‐24h groups compared to the control groups (*p* < 0.0001). In the chronic phase of RA in the TMJ (7 days post‐ intra‐articular injection), leukocyte levels significantly decreased and were comparable to those of the TMJ/C group, indicating that the inflammatory response was more pronounced in the early stage of arthritis (Figure 2a). In the knee, no significant difference was found between the K/RA‐24h and K/RA‐7d groups (Figure 2b).

**FIGURE 3:**
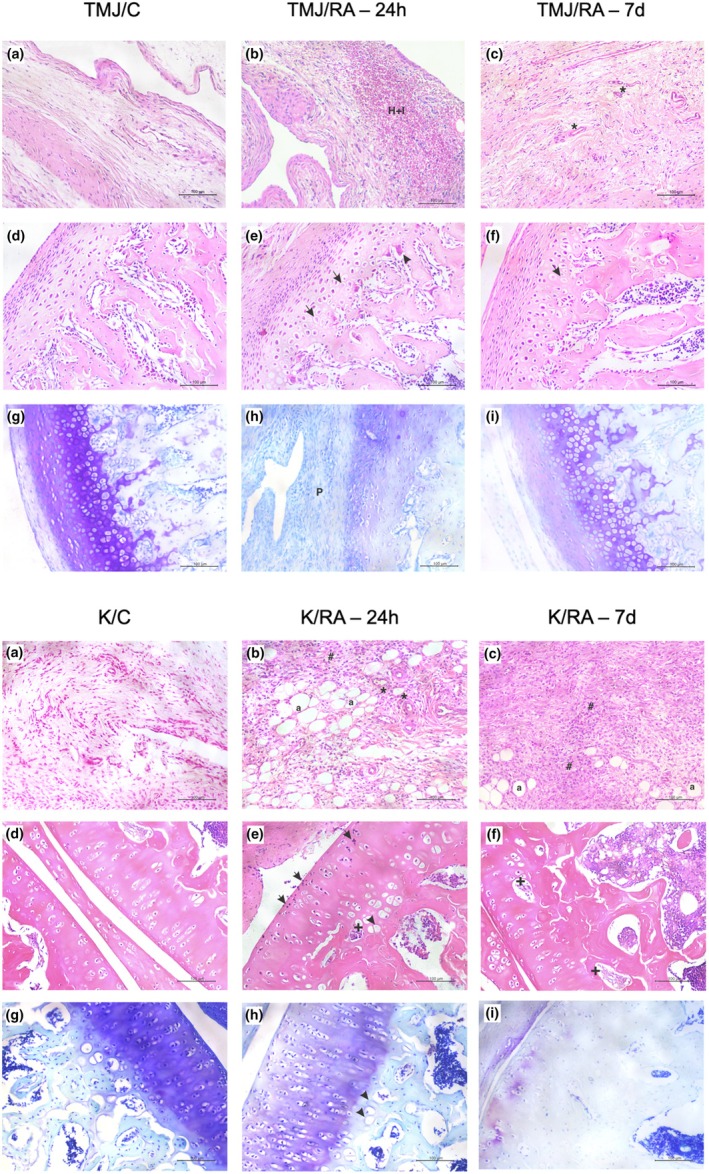
Photomicrographs of the TMJ and knee joints in arthritic animals. (a–c) Synovial membrane of animals with TMJ or knee arthritis (*n* = 6/ group; hematoxylin and eosin stain). (d–f) Articular cartilage of animals with TMJ or knee arthritis (*n* = 6/group; hematoxylin and eosin stain). (g–i) Articular cartilage of animals with TMJ or knee arthritis (*n* = 6/group; Toluidine Blue stain). TMJ = temporomandibular joint. TMJ/C = Control group; animals that did not receive mBSA in the TMJ. TMJ/RA‐24h = TMJ arthritic animals euthanized 24 h after the third intra‐articular injection of mBSA. TMJ/RA‐7d = TMJ arthritic animals euthanized 7 days after the third intra‐articular injection of mBSA. K/C = Control group; animals that did not receive mBSA in the knee joint. K/RA‐24h = Knee arthritic animals euthanized 24 h after the third intra‐articular injection of mBSA. K/RA‐7d = Knee arthritic animals euthanized 7 days after the third intra‐articular injection of mBSA. H + I = hemorrhage and inflammatory infiltrate. * = vascular neoformation. P = pannus. Arrow = hypertrophic and anucleated chondrocytes. Arrow tip = osteoclasts. Magnification 200x.

In the TMJ/RA‐24h group, histopathological analysis revealed edema, intense mononuclear inflammatory infiltrate, and hemorrhage in the synovial membrane, along with a reduction in intra‐articular space. Additionally, hypertrophic and anucleated chondrocytes were observed in the hypertrophic zone, as well as osteoclasts associated with empty lacunae. In contrast, the TMJ/RA‐7d group exhibited a reduction in these inflammatory parameters in both the synovial membrane and articular cartilage, suggesting a tissue repair response (Figure 3; Table 1).

**TABLE 1:** Semiquantitative analysis in the TMJ of arthritic rats.

Parameters	Control	RA‐24h	RA‐7d
Inflammatory infiltrate in the synovial membrane	0 (0–1)	3 (2–3)*	1 (0–2)
Damage to the articular cartilage and subchondral bone	0 (0–0)	2.5 (2–3)*	0.5 (0–1)
Presence of pannus	0 (0–0)	1 (0–1)*	0 (0–0)

*Note*: Control: Animals that did not receive mBSA in the TMJ; RA‐24h: TMJ arthritic animals euthanized 24 h after the third intra‐articular injection of mBSA; RA‐7d: TMJ arthritic animals euthanized 7 days after the third intra‐articular injection of mBSA.

* *p* < 0.05 versus Control (Kruskal–Wallis; Dunn).

In the knee joint, the K/RA‐24h and K/RA‐7d groups showed an intense mononuclear inflammatory infiltrate, vascular neoformation, and the presence of adipocytes in the synovial membrane. These findings were associated with hypertrophic and anucleated chondrocytes, foci of ossification, and migration of mononuclear inflammatory cells into the hyaline cartilage. In the K/RA‐7d group, a reduction in the number of chondrocytes, an increase in empty lacunae in the articular cartilage, and a reduction in intra‐articular space were observed (Figure 3; Table 2).

**TABLE 2:** Semiquantitative histopathological analysis in the knee joint of arthritic rats.

Parameters	Control	RA‐24h	RA‐7d
Inflammatory infiltrate in the synovial membrane	0 (0–1)	3 (2–3)*	2.5 (2–3)*
Damage to the articular cartilage and subchondral bone	0 (0–1)	2.5 (2–3)*	3 (2–3)*
Presence of pannus	0 (0–0)	1 (0–1)*	1 (0–1)*

*Note*: Control: Animals that did not receive mBSA in the knee joint; RA‐24h: Knee arthritic animals euthanized 24 h after the third intra‐articular injection of mBSA; RA‐7d: Knee arthritic animals euthanized 24 h after the third intra‐articular injection of mBSA.

* *p* < 0.05 versus Control (Kruskal–Wallis; Dunn).

**FIGURE 4:**
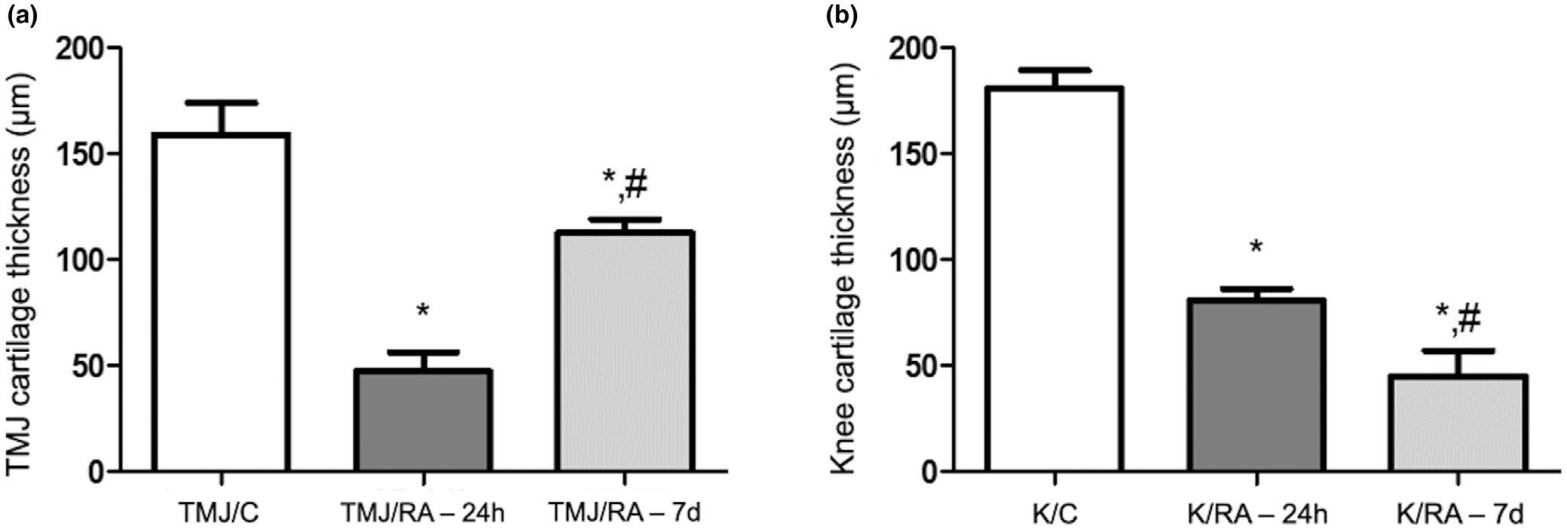
Articular cartilage thickness in the TMJ (a) or Knee (b) in rats with arthritis induced by mBSA (*n* = 6/group). TMJ = temporomandibular joint. Bars represent mean ± SD (One‐way ANOVA, Tukey). *p < 0.05 versus TMJ/C or K/C; #*p* < 0.05 versus TMJ/RA‐24h or K/RA‐24h. TMJ/C = Control group; animals that did not receive mBSA in the TMJ. TMJ/RA‐24h = TMJ arthritic animals euthanized 24 h after the third intra‐articular injection of mBSA. TMJ/RA‐7d = TMJ arthritic animals euthanized 7 days after the third intra‐articular injection of mBSA. K/C = Control group; animals that did not receive mBSA in the knee joint. K/RA‐24h = Knee arthritic animals euthanized 24 h after the third intra‐articular injection of mBSA. K/RA‐7d = Knee arthritic animals euthanized 7 days after the third intra‐articular injection of mBSA.

Toluidine blue staining of the TMJ revealed intense metachromasia and well‐organized histological layers of the articular cartilage (articular zone, zone of undifferentiated cells, flat zone, and hypertrophic zone) in the TMJ/C group (Figure 3g). In the TMJ/RA‐24h group, there was a marked reduction in metachromasia thickness and disorganization of the articular cartilage layers (Figures 3h and 4a). However, in the TMJ/RA‐7d group, the cartilage displayed increased thickness and metachromasia compared to the TMJ/RA‐24h group (*p* < 0.0001) (Figures 3i and 4a).

Regarding knee arthritis, the K/C group displayed intense metachromasia throughout the full thickness of the articular cartilage (Figure 4g). In the K/RA‐24h group, a reduction in cartilage thickness (*p* < 0.0001), metachromasia, and organization of the articular cartilage layers was observed compared to the K/C group (Figures 3h and 4b). In the K/RA‐7d group, the reduction in metachromasia was more pronounced, and differentiation between the hyaline and ossification zones was no longer distinguishable (Figure 3i).

### 3.3 Collagen fiber alterations in the articular cartilage of the TMJ and knee in rats with mBSA‐induced arthritis

**FIGURE 5:**
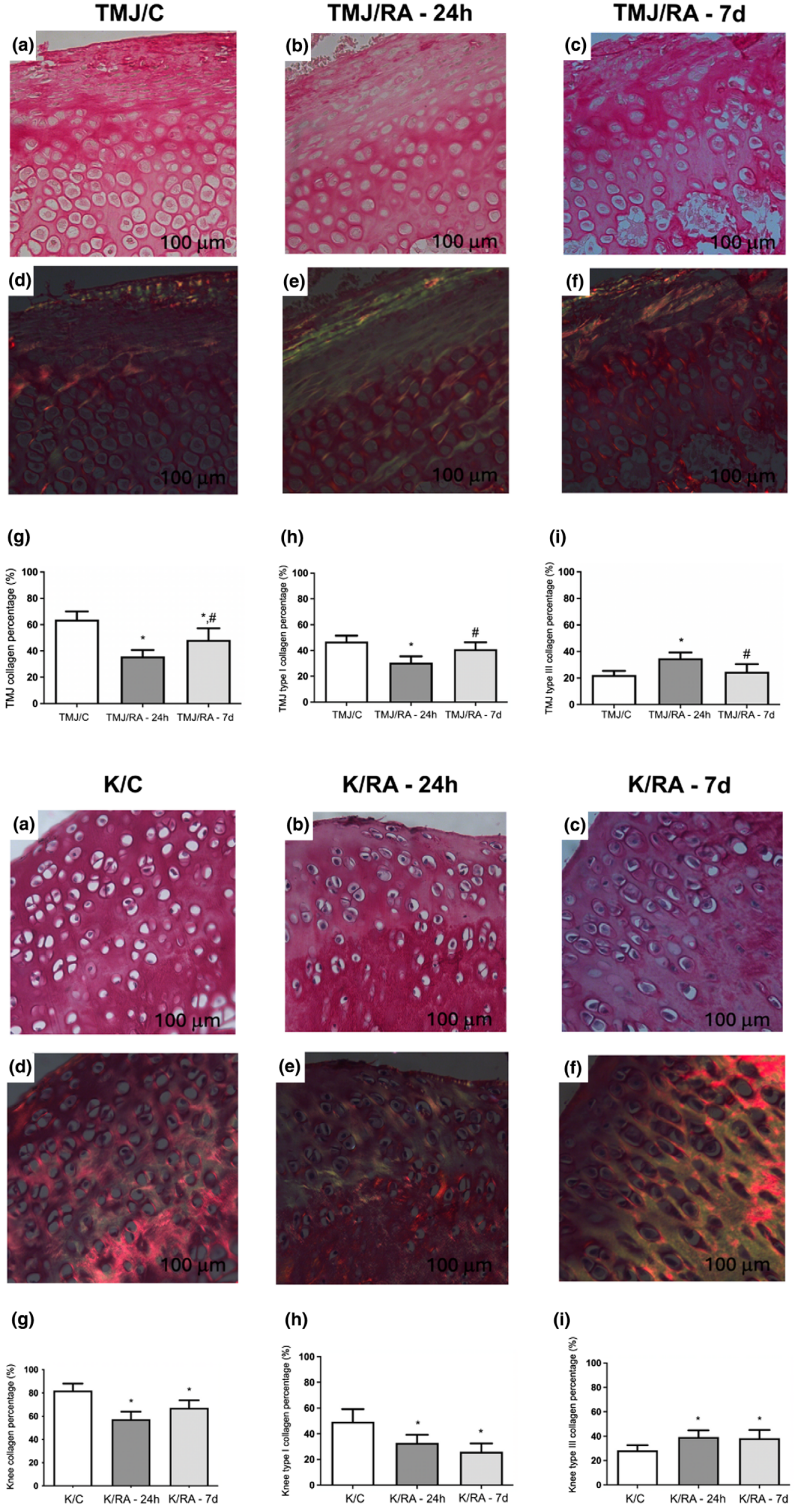
(a–c) Photomicrographs of articular cartilage in the TMJ or knee joint of arthritic rats. The articular cartilage of the TMJ or knee was stained with Picrosirius Red (d–f), and the total collagen (g) and type I (h) and III (i) collagen were quantified (*n* = 6/group). Bars represent mean ± SD (One‐way ANOVA, Tukey). *p < 0.05 versus TMJ/C or K/C; #*p* < 0.05 versus TMJ/RA‐24h. TMJ = temporomandibular joint. TMJ/C = Control group; animals that did not receive mBSA in the TMJ. TMJ/RA‐24h = TMJ arthritic animals euthanized 24 h after the third intra‐articular injection of mBSA. TMJ/RA‐7d = TMJ arthritic animals euthanized 7 days after the third intra‐articular injection of mBSA. K/C = Control group; animals that did not receive mBSA in the knee joint. K/RA‐24h = Knee arthritic animals euthanized 24 h after the third intra‐articular injection of mBSA. K/RA‐7d = Knee arthritic animals euthanized 7 days after the third intra‐articular injection of mBSA.

In the TMJ, the acute phase of the disease was characterized by a significant reduction in total collagen fibers (Figure 5g) and type I collagen (red‐orange fibers) (Figure 5h), along with an increase in type III collagen (greenish‐white fibers) (Figure 5i) compared to the TMJ/C group (*p* < 0.05). In the chronic phase, there was a significant increase in the percentage of total collagen fibers (Figure 5g) and type I collagen (Figure 5h), as well as a reduction in type III collagen (Figure 5i), compared to the RA‐24h group (*p* < 0.05).

In the knee, the acute‐phase response was similar to that observed in the TMJ. However, in the chronic phase, there was a significant decrease in the percentage of total collagen and type I collagen (red‐orange fibers) (*p* < 0.05; Figure 5g,h, respectively), along with a significant increase in the percentage of type III collagen (greenish‐white fibers) (*p* < 0.05; Figure 5i) compared to the K/C group.

### 3.4 MMP‐9 immunoexpression in the articular cartilage of the TMJ and knee

**FIGURE 6:**
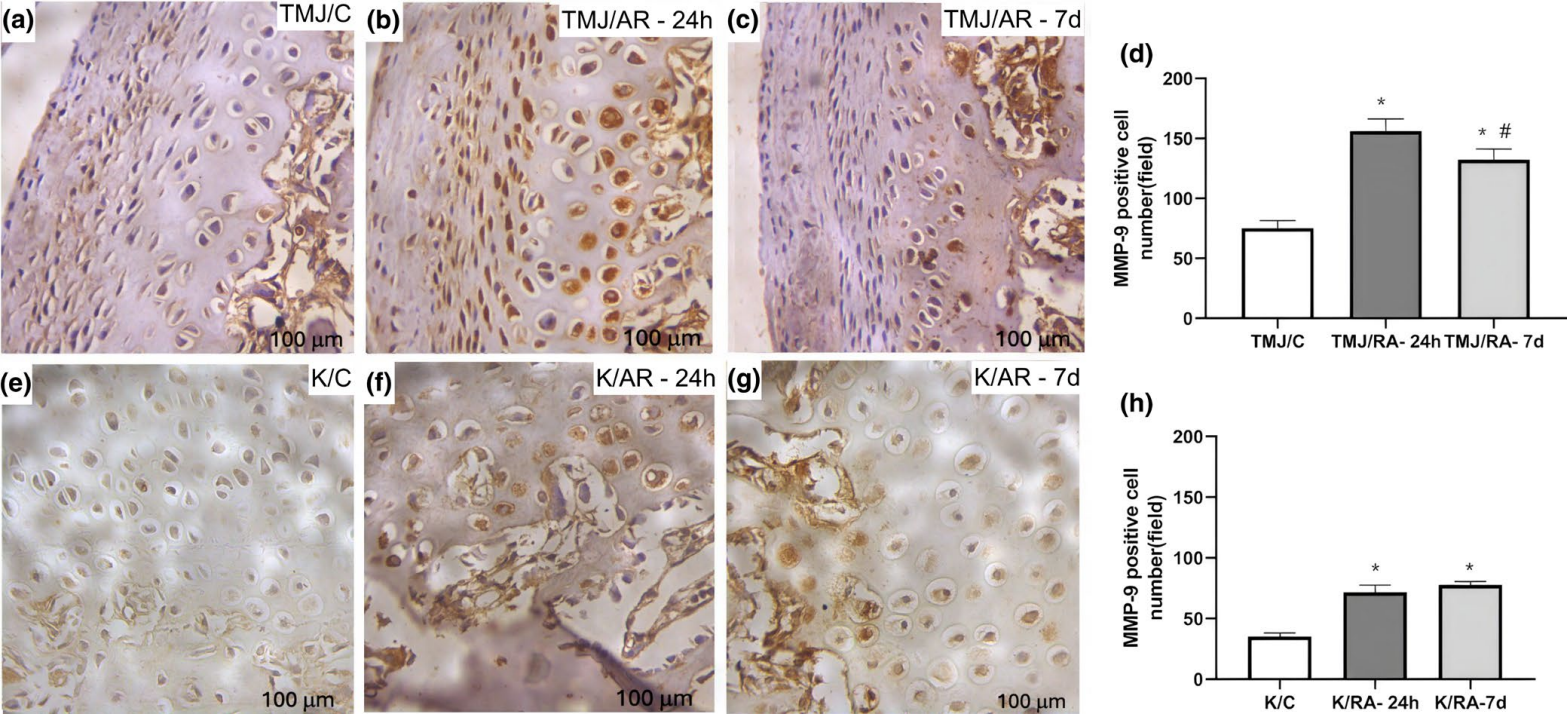
Photomicrographs of MMP‐9 immunoexpression in the articular cartilage of the TMJ (a–c) and knee joint (e–f) in arthritic rats. The number of MMP‐9‐positive cells in the TMJ (d) and knee joint (g) was quantified (*n* = 6/group). Bars represent mean ± SD (One‐way ANOVA, Tukey). *p < 0.05 versus TMJ/C or K/C; #*p* < 0.05 versus TMJ/RA‐24h. TMJ = temporomandibular joint. TMJ/C = Control group; animals that did not receive mBSA in the TMJ. TMJ/RA‐24h = TMJ arthritic animals euthanized 24 h after the third intra‐articular injection of mBSA. TMJ/RA‐7d = TMJ arthritic animals euthanized 7 days after the third intra‐articular injection of mBSA. K/C = Control group; animals that did not receive mBSA in the knee joint. K/RA‐24h = Knee arthritic animals euthanized 24 h after the third intra‐articular injection of mBSA. K/RA‐7d = Knee arthritic animals euthanized 7 days after the third intra‐articular injection of mBSA.

In the TMJ, the TMJ/RA‐24h and TMJ/RA‐7d groups showed a significant increase in MMP‐9 immunoexpression in the articular cartilage compared to the TMJ/C group (*p* < 0.0001). However, the TMJ/RA‐7d group exhibited a significant reduction in immunoexpression compared to the TMJ/RA‐24 h group (Figure 6d).

In the knee, MMP‐9 immunoexpression was significantly higher in arthritic animals (K/RA‐24h and K/RA‐7d) compared to non‐arthritic animals at both euthanasia time points (24 h and 7 days after the third mBSA injection). However, no significant difference was observed between the K/RA‐24h and K/RA‐7d groups (Figure 6h).

## 4 DISCUSSION

Our study conducted a comparative analysis of arthritis development induced by mBSA in two distinct synovial joints: the knee, which is covered by hyaline cartilage, and the TMJ, which is covered by fibrocartilage. In both joints, a significant reduction in the nociceptive threshold was observed during both the acute and chronic phases. However, while the inflammatory and morphological responses were similar in the acute phase, differences emerged in the chronic phase. Notably, the TMJ exhibited signs of recovery from the joint damage caused by intra‐articular mBSA injections, suggesting a repair process in this joint following injury.

Experimental models of joint inflammation are essential for gaining a deeper understanding of the molecular mechanisms and morphological impact underlying these conditions, as well as for developing more effective therapies (Leenaars et al., 2020). To the best of the authors' knowledge, this is the first study to compare the development of arthritis while considering the specific characteristics of the synovial joints in the TMJ and knee.

Most current knowledge of articular degradation in rheumatic diseases comes from studies on load‐bearing synovial joints, particularly the knee (Wang et al., 2023; Zhang et al., 2018). However, both the TMJ and knee are frequent sites of pain, inflammation, and restricted range of motion, even though the TMJ is rarely the first joint affected (Lin et al., 2020). Research indicates that more than half of RA patients exhibit clinical evidence of TMJ involvement, with 75% of these cases showing bilateral involvement (Mustafa et al., 2022). Temporomandibular symptoms typically emerge in more advanced stages of the disease and tend to worsen with movement and restricted mandibular mobility. Approximately 10% of RA patients develop permanent TMJ damage and occlusal changes (Mehra et al., 2009).

Regarding morphological changes, in general, RA is characterized by symmetrical synovitis, resulting in cartilage erosion and changes in bone anatomy. It is worth noting, however, that symptoms are variable during periods of exacerbation and remission of the disease (Buzatu & Moots, 2019). Synovitis is due to high levels of mononuclear inflammatory cells in synovial fluid, such as macrophages, monocytes, and lymphocytes, and high levels of pro‐inflammatory cytokines, such as IL‐1β, IL‐6, and TNF‐α (Jang et al., 2022).

During the inflammatory process of RA, the synovial membrane undergoes changes that promote inflammation, hemorrhage, alterations in joint fluid composition, and disruptions in cartilage and subchondral bone metabolism (Kronzer & Davis, 2021). In cartilage, chondrocyte hypertrophy and cell death are common features of RA progression (Li et al., 2021; Rim et al., 2020). Some of these cells actively proliferate, increase in volume, and become mineralized. As a result, the cartilage loses its elasticity and becomes stiffer (Blanco et al., 2021).

As synovial membrane hyperplasia progresses, macrophage and lymphocyte infiltration, as well as angiogenesis, are observed. This is accompanied by the release of pro‐inflammatory cytokines, growth factors, and proteolytic enzymes that contribute to joint damage. Additionally, this inflammatory state stimulates adipocyte production within the synovial membrane (Sudoł‐Szopińska et al., 2013). Adipose tissue is now recognized as a specialized form of connective tissue and an endocrine organ, meaning its presence can influence synovial metabolism (Kuca‐Warnawin et al., 2023). When synovitis develops, the synovial tissue undergoes pathological fibrosis due to excessive extracellular matrix (ECM) deposition, primarily mediated by synovial fibroblasts (Elhaj Mahmoud et al., 2021).

In the TMJ, the ECM has a unique hybrid structure, consisting of a fibrocartilage layer covering secondary hyaline cartilage (Hinton, 2014). This differs from other joint surfaces, which are composed solely of primary hyaline cartilage. The ECM of the TMJ is derived from the ectodermal neural crest and develops relatively late in embryogenesis, whereas the knee's ECM originates from the mesodermal chondroskeleton (Chandrasekaran et al., 2021). The molecular mechanisms involved in TMJ ECM formation remain poorly understood, but fibrocartilage is thought to contribute to the joint's greater repair capacity (Govind et al., 2023). Our study supports this idea, as the TMJ/RA‐7d group exhibited improvements in histopathological changes related to inflammation and joint damage, whereas in the K/RA‐7d group, joint injury persisted.

The release of proteinases in the ECM, specifically matrix metalloproteinases (MMPs), also contributes to joint degeneration. MMPs are a family of 20 structurally related proteolytic enzymes that are secreted as inactive proenzymes and become activated primarily in the extracellular environment. These enzymes collectively degrade proteoglycans, collagen, and other ECM components (Cho et al., 2019).

MMPs are classified into subgroups based on their substrate specificity: collagenases (MMP‐1, MMP‐8, and MMP‐13) degrade fibrillar collagens of types I, II, and III; stromelysins (MMP‐3 and MMP‐11) degrade proteoglycans and non‐helical regions of collagen; and gelatinases (MMP‐2 and MMP‐9) have high specificity for degraded collagen (Shibata et al., 1998). Notably, TMJ fibrocartilage predominantly consists of type I collagen, whereas knee hyaline cartilage is mainly composed of type II collagen (Gepstein et al., 2002).

The evaluation of proteoglycans in this study revealed a marked reduction in metachromasia and disorganization of the articular cartilage layers in both the TMJ and knee of arthritic rats. Proteoglycan depletion plays a crucial role in articular cartilage destruction, working in conjunction with MMPs to stimulate processes such as type I collagen degradation (Bopp‐ Kuchler et al., 2020). Consistent with this, our study found an increase in MMP‐9 immunoexpression alongside a reduction in total collagen fibers and type I collagen in arthritic animals during the acute phase of the disease compared to healthy controls. However, there was an increase in type III collagen in the acute phase in both joints. In the chronic phase, joint damage persisted in the knee, whereas in the TMJ, there was an improvement in these ECM degradation‐related parameters, suggesting a recovery process in this joint.

Pain is a significant concern in RA, and during the acute phase, it is primarily linked to local joint inflammation (Bas et al., 2012; Salaffi et al., 2019; Williams et al., 2020; Zhang & Lee, 2018). However, in the chronic phase, pain may also be influenced by neuroimmune interactions and non‐inflammatory mechanisms, such as neuroglial interactions or central pain processing (Totsch & Sorge, 2017). As a result, persistent pain during RA remission remains a major challenge for patients (Süß et al., 2020).

Our findings indicate that in knee arthritis, pain and joint damage are present and correlated in both the acute and chronic phases. However, in the TMJ, the chronic stage of the disease demonstrated joint repair following the damage observed in the acute phase. Nonetheless, animals exhibited lower nociceptive thresholds, suggesting the presence of central sensitization despite the observed tissue recovery over time.

These findings suggest that pain management strategies for TMJ arthritis may need to differ from those used for other synovial joints. It is possible that patients with chronic TMJ arthritis could benefit more from centrally acting therapies, given that no peripheral joint damage was observed.

A limitation of this study is that it may not fully replicate the complexity of human RA. Moreover, additional functional and molecular assessments would be valuable to provide a more comprehensive understanding of the mechanisms underlying the differences observed between joints.

Based on these insights, future studies should focus on investigating the immune mechanisms and signaling pathways involved in RA pathogenesis in the TMJ and other synovial joints. A deeper understanding of these processes may contribute to the development of more effective therapies tailored to the specific responses of each joint to inflammatory disease.

In summary, the development of arthritis in the TMJ and knee joint follows a similar pattern during the acute phase of the disease. However, in the chronic phase, arthritic rats with TMJ involvement exhibited increased pain sensitivity even in the absence of inflammation and joint damage, suggesting a process of central pain sensitization. This finding highlights the potential need to adjust therapeutic approaches for chronic TMJ arthritis to better address pain mechanisms specific to this joint.

## AUTHOR CONTRIBUTIONS

A.C.F.C., L.M.S., and D.V.G. participated in the conceptualization and design of the study. A.C.F.C. and L.M.S. were responsible for the investigation, data collection, and data analysis. A.C.F.C. and D.V.G. drafted the manuscript, and all authors coedited and approved the final version.

## CONFLICT OF INTEREST STATEMENT

The authors declare no conflict of interest.

## Supporting information


**Data S1.** Experimental protocol. mBSA, methylated bovine serum albumin; CFA, complete Freund’s adjuvant; IFA, incomplete Freund’s adjuvant.

## Data Availability

Data will be made available upon request.

## References

[joa70080-bib-0001] Almutairi, K. , Nossent, J. , Preen, D. , Keen, H. & Inderjeeth, C. (2021) The global prevalence of rheumatoid arthritis: a meta‐analysis based on a systematic review. Rheumatology International, 41(5), 863–877. 33175207 10.1007/s00296-020-04731-0

[joa70080-bib-0003] Bas, D.B. , Su, J. , Sandor, K. , Agalave, N.M. , Lundberg, J. , Codeluppi, S. et al. (2012) Collagen antibody–induced arthritis evokes persistent pain with spinal glial involvement and transient prostaglandin dependency. Arthritis and Rheumatism, 64(12), 3886–3896. 22933386 10.1002/art.37686

[joa70080-bib-0004] Blanco, M.F. , Garcia, H.D. , Legeai‐Mallet, L. & van Osch, G.J. (2021) Tyrosine kinases regulate chondrocyte hypertrophy: promising drug targets for osteoarthritis. Osteoarthritis and Cartilage, 29(10), 1389–1398. 34284112 10.1016/j.joca.2021.07.003

[joa70080-bib-0005] Bopp‐ Kuchler, S. , Mariotte, A. , Strub, M. , Po, C. , de Cauwer, A. , Schulz, G. et al. (2020) Temporomandibular joint damage in K/BxN arthritic mice. International Journal of Oral Science, 12(1), 5. 32024813 10.1038/s41368-019-0072-z PMC7002582

[joa70080-bib-0006] Buzatu, C. & Moots, R.J. (2019) Measuring disease activity and response to treatment in rheumatoid arthritis. Expert Review of Clinical Immunology, 15(2), 135–145. 30556738 10.1080/1744666X.2019.1559050

[joa70080-bib-0007] Camargo, L.L. , Denadai‐Souza, A. , Yshii, L.M. , Lima, C. , Teixeira, S.A. , Cerqueira, A.R. et al. (2021) The potential anti‐inflammatory and anti‐nociceptive effects of rat hemopressin (PVNFKFLSH) in experimental arthritis. European Journal of Pharmacology, 890, 173636. 33053380 10.1016/j.ejphar.2020.173636

[joa70080-bib-0008] Chandrasekaran, P. , Kwok, B. , Han, B. , Adams, S.M. , Wang, C. , Chery, D.R. et al. (2021) Type V collagen regulates the structure and biomechanics of TMJ condylar cartilage: a fibrous‐hyaline hybrid. Matrix Biology, 102, 1–19. 34314838 10.1016/j.matbio.2021.07.002 PMC8549065

[joa70080-bib-0010] Chaurasia, N. , Singh, A. , Singh, I.L. , Singh, T. & Tiwari, T. (2020) Cognitive dysfunction in patients of rheumatoid arthritis. Journal of Family Medicine and Primary Care, 9(5), 2219–2225. 10.4103/jfmpc.jfmpc_307_20 PMC7380780 32754477

[joa70080-bib-0011] Cho, C.H. , Lho, Y.M. , Hwang, I. & Kim, D.H. (2019) Role of matrix metalloproteinases 2 and 9 in the development of frozen shoulder: human data and experimental analysis in a rat contracture model. Journal of Shoulder and Elbow Surgery, 28(7), 1265–1272. 30846222 10.1016/j.jse.2018.11.072

[joa70080-bib-0012] Conforti, A. , Di Cola, I. , Pavlych, V. , Ruscitti, P. , Berardicurti, O. , Ursini, F. et al. (2021) Beyond the joints, the extra‐articular manifestations in rheumatoid arthritis. Autoimmunity Reviews, 20(2), 102735. 33346115 10.1016/j.autrev.2020.102735

[joa70080-bib-0013] de Sousa, L.M. , dos Santos Alves, J.M. , da Silva Martins, C. , Pereira, K.M.A. , Goes, P. & Gondim, D.V. (2019) Immunoexpression of canonical Wnt and NF‐κB signaling pathways in the temporomandibular joint of arthritic rats. Inflammation Research, 68, 889–900. 31372663 10.1007/s00011-019-01274-4

[joa70080-bib-0014] de Sousa, L.M. , Figueiredo Costa, A.C. , Pereira, A.F. , da Silva Martins, C. , de Oliveira Filho, O.V. , Goes, P. et al. (2023) Temporomandibular joint arthritis increases canonical Wnt pathway expression in the articular cartilage and trigeminal ganglion in rats. Bone Reports, 18, 101649. 36700243 10.1016/j.bonr.2022.101649 PMC9869417

[joa70080-bib-0015] de Sousa, V.C. , Sousa, F.R.N. , Vasconcelos, R.F. , Martins, C.S. , Lopes, A.P. , Alves, N.M. et al. (2022) Atorvastatin reduces zoledronic acid‐induced osteonecrosis of the jaws of rats. Bone, 164, 116523. 35985466 10.1016/j.bone.2022.116523

[joa70080-bib-0016] Elhaj Mahmoud, D. , Kaabachi, W. , Sassi, N. , Mokhtar, A. , Ben Ammar, L. , Rekik, S. et al. (2021) Expression of extracellular matrix components and cytokine receptors in human fibrocytes during rheumatoid arthritis. Connective Tissue Research, 62(6), 720–731. 33427511 10.1080/03008207.2021.1873962

[joa70080-bib-0017] Fazaeli, S. , Ghazanfari, S. , Everts, V. , Smit, T.H. & Koolstra, J.H. (2016) The contribution of collagen fibers to the mechanical compressive properties of the temporomandibular joint disc. Osteoarthritis and Cartilage, 24(7), 1292–1301. 26828357 10.1016/j.joca.2016.01.138

[joa70080-bib-0018] Fraenkel, L. , Bathon, J.M. , England, B.R. , St. Clair, E.W. , Arayssi, T. , Carandang, K. et al. (2021) 2021 American College of Rheumatology guideline for the treatment of rheumatoid arthritis. Arthritis and Rheumatology, 73(7), 1108–1123. 34101376 10.1002/art.41752

[joa70080-bib-0019] Gepstein, A. , Shapiro, S. , Arbel, G. , Lahat, N. & Livne, E. (2002) Expression of matrix metalloproteinases in articular cartilage of temporomandibular and knee joints of mice during growth, maturation, and aging. Arthritis & Rheumatism, 46(12), 3240–3250. 12483728 10.1002/art.10690

[joa70080-bib-0020] Govind, A. , Ploussard, D. , Hoffman, S. , Zhang, Z. & Wu, Y. (2023) Medicare coverage patterns favor non‐invasive and minimally‐invasive treatments of knee osteoarthritis compared to temporomandibular joint osteoarthritis. Cranio, 41(2), 144–150. 32991257 10.1080/08869634.2020.1826174

[joa70080-bib-0021] Gusmão, J.N.F.M. , Fonseca, K.M. , Ferreira, B.S.P. , de Freitas Alves, B.W. , Ribeiro Júnior, H.L. , Lisboa, M.R.P. et al. (2021) Electroacupuncture reduces inflammation but not bone loss on periodontitis in arthritic rats. Inflammation, 44(1), 116–128. 32789781 10.1007/s10753-020-01313-x

[joa70080-bib-0022] Gutman, S. , Kim, D. , Tarafder, S. , Velez, S. , Jeong, J. & Lee, C.H. (2018) Regionally variant collagen alignment correlates with viscoelastic properties of the disc of the human temporomandibular joint. Archives of Oral Biology, 86, 1–6. 29128675 10.1016/j.archoralbio.2017.11.002 PMC5745264

[joa70080-bib-0023] Hinton, R.J. (2014) Genes that regulate morphogenesis and growth of the temporomandibular joint: a review. Developmental Dynamics, 243(7), 864–874. 24668501 10.1002/dvdy.24130

[joa70080-bib-0024] Jang, S. , Kwon, E.J. & Lee, J.J. (2022) Rheumatoid arthritis: pathogenic roles of diverse immune cells. International Journal of Molecular Sciences, 23(2), 905. 35055087 10.3390/ijms23020905 PMC8780115

[joa70080-bib-0026] Kronzer, V.L. & Davis, J.M. (2021) Etiologies of rheumatoid arthritis: update on mucosal, genetic, and cellular pathogenesis. Current Rheumatology Reports, 23, 1–10. 10.1007/s11926-021-00993-0 PMC7919619 33646410

[joa70080-bib-0027] Kubiak, M. & Ditzel, M. (2016) A joint less ordinary: intriguing roles for hedgehog signalling in the development of the temporomandibular synovial joint. Journal of Developmental Biology, 4(3), 25. 29615589 10.3390/jdb4030025 PMC5831777

[joa70080-bib-0028] Kuca‐Warnawin, E. , Kurowska, W. , Plebańczyk, M. , Wajda, A. , Kornatka, A. , Burakowski, T. et al. (2023) Basic properties of adipose‐derived mesenchymal stem cells of rheumatoid arthritis and osteoarthritis patients. Pharmaceutics, 15(3), 1003. 36986863 10.3390/pharmaceutics15031003 PMC10051260

[joa70080-bib-0029] Kurowska, W. , Kuca‐Warnawin, E.H. , Radzikowska, A. & Maśliński, W. (2017) The role of anti‐citrullinated protein antibodies (ACPA) in the pathogenesis of rheumatoid arthritis. Central European Journal of Immunology, 42(4), 390–398. 29472818 10.5114/ceji.2017.72807 PMC5820977

[joa70080-bib-0030] Lassere, M.N. , Rappo, J. , Portek, I.J. , Sturgess, A. & Edmonds, J.P. (2013) How many life years are lost in patients with rheumatoid arthritis? Secular cause‐specific and all‐cause mortality in rheumatoid arthritis, and their predictors in a long‐term a ustralian cohort study. Internal Medicine Journal, 43(1), 66–72. 22289054 10.1111/j.1445-5994.2012.02727.x

[joa70080-bib-0031] Leenaars, C. , Stafleu, F. , de Jong, D. , van Berlo, M. , Geurts, T. , Coenen‐de Roo, T. et al. (2020) A systematic review comparing experimental design of animal and human methotrexate efficacy studies for rheumatoid arthritis: lessons for the translational value of animal studies. Animals, 10(6), 1047. 32560528 10.3390/ani10061047 PMC7341304

[joa70080-bib-0032] Lemos, G.A. , da Silva, P.L.P. , Batista, A.U.D. & Palomari, E.T. (2018) Experimental model of temporomandibular joint arthritis: evaluation of contralateral joint and masticatory muscles. Archives of Oral Biology, 95, 79–88. 30071410 10.1016/j.archoralbio.2018.07.003

[joa70080-bib-0033] Li, B. , Guan, G. , Mei, L. , Jiao, K. & Li, H. (2021) Pathological mechanism of chondrocytes and the surrounding environment during osteoarthritis of temporomandibular joint. Journal of Cellular and Molecular Medicine, 25(11), 4902–4911. 33949768 10.1111/jcmm.16514 PMC8178251

[joa70080-bib-0034] Lin, Y.J. , Anzaghe, M. & Schülke, S. (2020) Update on the pathomechanism, diagnosis, and treatment options for rheumatoid arthritis. Cells, 9(4), 880. 32260219 10.3390/cells9040880 PMC7226834

[joa70080-bib-0035] Mehra, P. , Wolford, L.M. , Baran, S. & Cassano, D.S. (2009) Single‐stage comprehensive surgical treatment of the rheumatoid arthritis temporomandibular joint patient. Journal of Oral and Maxillofacial Surgery, 67(9), 1859–1872. 19686922 10.1016/j.joms.2009.04.035

[joa70080-bib-0036] Mustafa, M.A. , Al‐Attas, B.A. , Badr, F.F. , Jadu, F.M. , Wali, S.O. , Bawazir, Y.M. et al. (2022) Prevalence and severity of temporomandibular disorders in rheumatoid arthritis patients. Cureus, 14(1), e21276. 35070578 10.7759/cureus.21276 PMC8761059

[joa70080-bib-0037] Nazet, U. , Neubert, P. , Schatz, V. , Grässel, S. , Proff, P. , Jantsch, J. et al. (2022) Differential gene expression response of synovial fibroblasts from temporomandibular joints and knee joints to dynamic tensile stress. Journal of Orofacial Orthopedics / Fortschritte der Kieferorthopädie, 83(6), 361–375. 34142176 10.1007/s00056-021-00309-y PMC9596579

[joa70080-bib-0038] Quinteiro, M.S. , Napimoga, M.H. , Macedo, C.G. , Freitas, F.F. , Abdalla, H.B. , Bonfante, R. et al. (2014) 15‐deoxy‐Δ12,14‐prostaglandin J2 reduces albumin‐induced arthritis in temporomandibular joint of rats. European Journal of Pharmacology, 740, 58–65. 25016088 10.1016/j.ejphar.2014.07.002

[joa70080-bib-0039] Radu, A.F. & Bungau, S.G. (2021) Management of rheumatoid arthritis: an overview. Cells, 10(11), 2857. 34831081 10.3390/cells10112857 PMC8616326

[joa70080-bib-0040] Rim, Y.A. , Nam, Y. & Ju, J.H. (2020) The role of chondrocyte hypertrophy and senescence in osteoarthritis initiation and progression. International Journal of Molecular Sciences, 21(7), 2358. 32235300 10.3390/ijms21072358 PMC7177949

[joa70080-bib-0041] Salaffi, F. , Di Carlo, M. , Carotti, M. & Sarzi‐Puttini, P. (2019) The effect of neuropathic pain symptoms on remission in patients with early rheumatoid arthritis. Current Rheumatology Reviews, 15(2), 154–161. 30081788 10.2174/1573397114666180806142814

[joa70080-bib-0042] Scherer, H.U. , Häupl, T. & Burmester, G.R. (2020) The etiology of rheumatoid arthritis. Journal of Autoimmunity, 110, 102400. 31980337 10.1016/j.jaut.2019.102400

[joa70080-bib-0043] Shibata, T. , Murakami, K.I. , Kubota, E. & Maeda, H. (1998) Glycosaminoglycan components in temporomandibular joint synovial fluid as markers of joint pathology. Journal of Oral and Maxillofacial Surgery, 56(2), 209–213. 9461147 10.1016/s0278-2391(98)90871-0

[joa70080-bib-0044] Smolen, J.S. , Aletaha, D. , Bijlsma, J.W. , Breedveld, F.C. , Boumpas, D. , Burmester, G. et al. (2010) Treating rheumatoid arthritis to target: recommendations of an international task force. Annals of the Rheumatic Diseases, 69(4), 631–637. 20215140 10.1136/ard.2009.123919 PMC3015099

[joa70080-bib-0045] Sudoł‐Szopińska, I. , Kontny, E. , Zaniewicz‐Kaniewska, K. , Prohorec‐Sobieszek, M. , Saied, F. & Maśliński, W. (2013) Role of inflammatory factors and adipose tissue in pathogenesis of rheumatoid arthritis and osteoarthritis. Part I: rheumatoid adipose tissue. Journal of Ultrasonography, 13(53), 192. 26674614 10.15557/JoU.2013.0019 PMC4613583

[joa70080-bib-0046] Süß, P. , Rothe, T. , Hoffmann, A. , Schlachetzki, J.C. & Winkler, J. (2020) The joint‐brain axis: insights from rheumatoid arthritis on the crosstalk between chronic peripheral inflammation and the brain. Frontiers in Immunology, 11, 612104. 33362800 10.3389/fimmu.2020.612104 PMC7758283

[joa70080-bib-0054] Tak, P.P. , Smeets, T.J. , Daha, M.R. , Kluin, P.M. , Meijers, K.A. , Brand, R. et al. (1997) Analysis of the synovial cell infiltrate in early rheumatoid synovial tissue in relation to local disease activity. Arthritis and Rheumatology, 40(2), 217–225. 10.1002/art.1780400206 9041933

[joa70080-bib-0047] Totsch, S.K. & Sorge, R.E. (2017) Immune system involvement in specific pain conditions. Molecular Pain, 13, 1744806917724559. 28741433 10.1177/1744806917724559 PMC5555497

[joa70080-bib-0048] Vos, L.M. , Kuijer, R. , Slater, J.J.H. , Bulstra, S.K. & Stegenga, B. (2014) Inflammation is more distinct in temporomandibular joint osteoarthritis compared to the knee joint. Journal of Oral and Maxillofacial Surgery, 72(1), 35–40. 24210930 10.1016/j.joms.2013.08.022

[joa70080-bib-0049] Wang, S. , Zhou, Y. , Huang, J. , Li, H. , Pang, H. , Niu, D. et al. (2023) Advances in experimental models of rheumatoid arthritis. European Journal of Immunology, 53(1), 2249962. 10.1002/eji.202249962 36330559

[joa70080-bib-0050] Williams, B. , Lees, F. , Tsangari, H. , Hutchinson, M.R. , Perilli, E. & Crotti, T.N. (2020) Assessing the effects of parthenolide on inflammation, bone loss, and glial cells within a collagen antibody‐induced arthritis mouse model. Mediators of Inflammation, 2020, 6245798. 32189995 10.1155/2020/6245798 PMC7073477

[joa70080-bib-0051] Zhang, A. & Lee, Y.C. (2018) Mechanisms for joint pain in rheumatoid arthritis (RA): from cytokines to central sensitization. Current Osteoporosis Reports, 16, 603–610. 30128836 10.1007/s11914-018-0473-5 PMC6157001

[joa70080-bib-0053] Zhang, Q. , Dehaini, D. , Zhang, Y. , Zhou, J. , Chen, X. , Zhang, L. et al. (2018) Neutrophil membrane‐coated nanoparticles inhibit synovial inflammation and alleviate joint damage in inflammatory arthritis. Nature Nanotechnology, 13(12), 1182–1190. 10.1038/s41565-018-0254-4 30177807

