# Supplementary material for: Is the temporomandibular joint affected by rheumatoid arthritis? A comparative investigation with knee arthritis in an experimental rat model
Source: J Anat. 2025 Dec 7;249(1):114–26. doi: 10.1111/joa.70080 (PMC13238891; doi:10.1111/joa.70080)
Supplement: Supplementary file 1 — Data S1. Experimental protocol. mBSA, methylated bovine serum albumin; CFA, complete Freund’s adjuvant; IFA, incomplete Freund’s adjuvant. [file JOA-249-114-s001.docx]

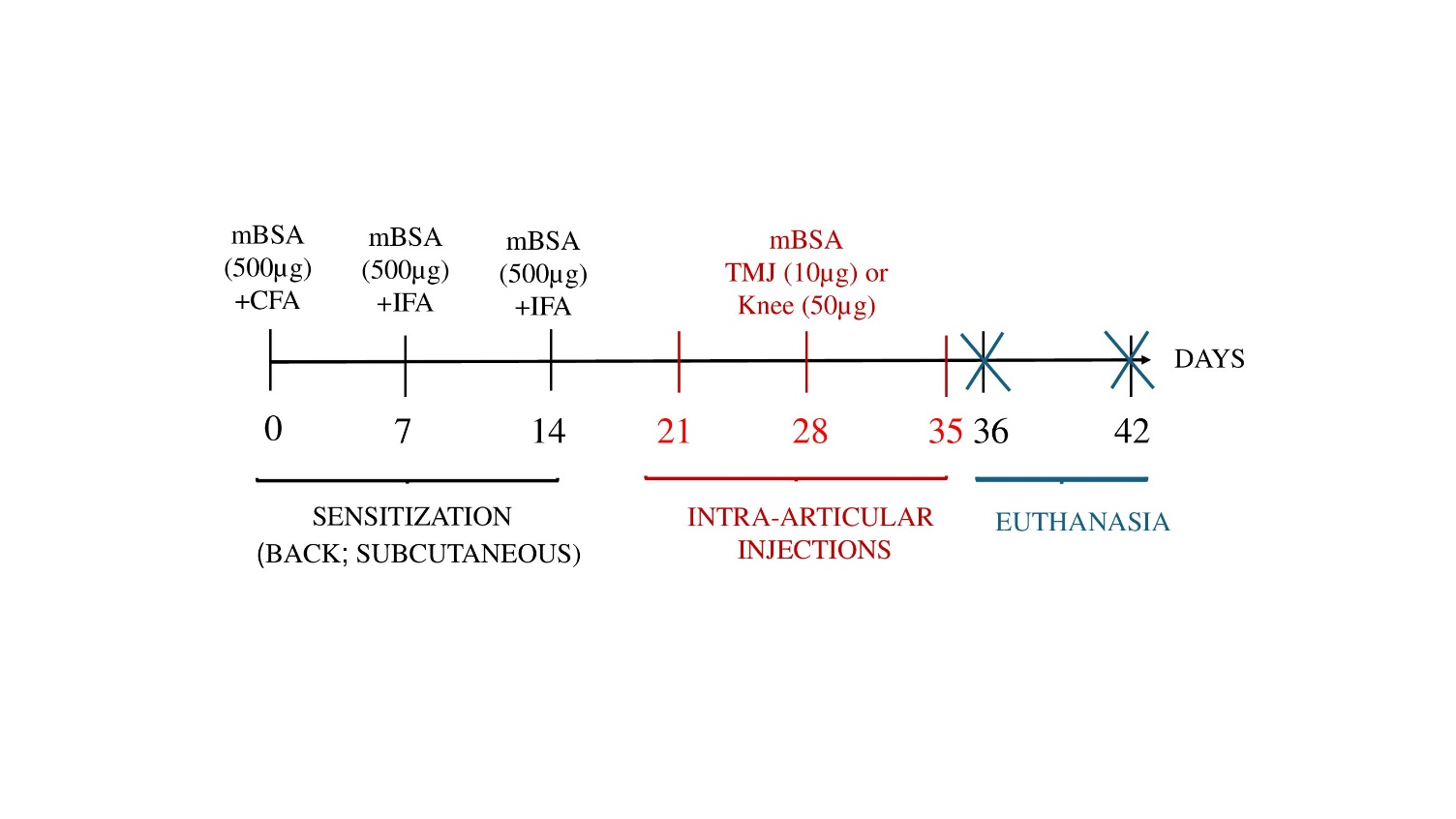
Supplementary Material 1. Experimental protocol. mBSA= methylated bovine serum albumin. CFA= Complete Freund’s Adjuvant. IFA= Incomplete Freund’s Adjuvant.
